# Biomarker Testing Patterns and Treatment Outcomes in Patients With Advanced Non-Small Cell Lung Cancer and *MET* Exon 14 Skipping Mutations: A Descriptive Analysis From the US

**DOI:** 10.3389/fonc.2022.786124

**Published:** 2022-02-25

**Authors:** Fatemeh Asad Zadeh Vosta Kolaei, Beilei Cai, Hemanth Kanakamedala, Julia Kim, Vitalii Doban, Shiyu Zhang, Michael Shi

**Affiliations:** ^1^ Novartis Pharmaceuticals Corporation, East Hanover, NJ, United States; ^2^ Genesis Research, Hoboken, NJ, United States

**Keywords:** next-generation sequencing, PD-L1, chemotherapy, immuno-oncology therapy, real world outcomes

## Abstract

**Background:**

*MET* exon 14 skipping mutation (*MET*ex14) is observed in ~3% of non-small cell lung cancer (NSCLC) cases and has been shown to be an independent poor prognostic factor associated with shorter overall disease-specific survival. Broad molecular testing can identify this biomarker in patients with advanced NSCLC (aNSCLC) and allow patients to be matched with the appropriate targeted therapy. This study examines biomarker testing patterns and clinical outcomes of chemotherapy and immuno-oncology (IO) monotherapy in aNSCLC patients with *MET*ex14.

**Methods:**

A descriptive retrospective study was conducted using the Flatiron Health–Foundation Medicine Inc. (FMI) clinico-genomic database. Patients with *MET*ex14 aNSCLC treated with systemic therapies were included in the biomarker testing analysis. The duration from specimen collection to reported results was assessed for PD-L1– and *MET*ex14-tested patients. Clinical outcomes were assessed in patients treated with chemotherapy or IO monotherapy. First-line (1L) and second-line (2L) real-world progression-free survival (rw-PFS) were estimated using Kaplan-Meier analysis.

**Results:**

Of 91 *MET*ex14 patients eligible for the biomarker testing analysis, 77% and 60% received PD-L1 and FMI next-generation sequencing (NGS) testing within 3 months post aNSCLC diagnosis. Of those assessed for both PD-L1 and *MET*ex14 (n=9), the median duration between specimen collection and reporting was 1 week shorter for PD-L1 than for FMI NGS. Median 1L rw-PFS was 5.7 months (95% CI, 4.6-7.1) and 2.4 months (95% CI, 1.4-3.2) in patients receiving 1L chemotherapy (n=59) and IO monotherapy (n=18), with 3-month 1L rw-PFS rates of 78% and 33%. Median 2L rw-PFS was 3.5 months (95% CI, 1.9-11.1) and 4.7 months (95% CI, 2.8-12.9) in patients receiving 2L chemotherapy (n=16) and IO monotherapy (n=23), with 3-month 2L rw-PFS rates of 54% and 67%.

**Conclusions:**

The median time from biopsy to test results appears 1 week shorter for PD-L1 than for FMI NGS. Chemotherapy and IO monotherapy were the most common regimens utilized but with limited PFS.

## Introduction

Non-small cell lung cancer (NSCLC), which accounts for ~85% of lung cancer diagnoses, is a heterogeneous disease consisting of multiple histologies and many known driver mutations (1–4). *MET* exon 14 skipping mutation (*MET*ex14) is observed in ~3% (95% CI, 1%-4%) of NSCLC cases; is more likely to occur in females, elderly patients (median age ranging from 65 to 76 years old), and nonsmokers (5); and typically occurs in the absence of other driver mutations (2, 3, 6). In retrospective studies of patients with resected NSCLC, *MET*ex14 has been shown to be an independent poor prognostic factor and is associated with shorter overall survival (7, 8), underscoring the need for identification of this biomarker in patients with NSCLC and for targeted therapies (9).

Capmatinib was approved by the US Food and Drug Administration (FDA) on May 6, 2020, for the treatment of adult patients with metastatic NSCLC whose tumors have a mutation that leads to *MET*ex14. Approval was based on results from the phase 2 GEOMETRY mono-1 trial (NCT02414139) (10, 11). In the trial, a blinded independent review committee confirmed an overall response rate (ORR) of 68% (95% CI, 48%-84%) and median duration of response (DOR) of 12.6 months (95% CI, 5.6 months-not estimable [NE]) among treatment-naive patients (N=28). An ORR of 41% (95% CI, 29%-53%) and median DOR of 9.7 months (95% CI, 5.6-13.0 months) were established among previously treated patients (N=69) (11).

Additionally, capmatinib has shown clinical evidence of intracranial activity. In the GEOMETRY mono-1 trial, among 13 patients who had data evaluable by an independent neuroradiologic review committee, 12 (92%) had intracranial disease control and 7 (54%) had an intracranial response. Of the patients with an intracranial response, 4 (57%) had a complete response (11).

Tepotinib, another MET inhibitor, was approved by the FDA on February 3, 2021, for the treatment of adult patients with metastatic NSCLC harboring *MET*ex14 mutations. Approval was based on results from the phase 2 VISION trial (NCT02864992) (12, 13). Among patients with *MET*ex14 NSCLC treated with tepotinib, the ORR was 46% (95% CI, 36%-57%) and the median DOR was 11.1 months (95% CI, 7.2 months-NE), according to independent review (12). In the 11 patients with brain metastases, the response rate by independent review was 55% (95% CI, 23%-83%), with a median DOR of 9.5 months (95% CI, 6.6 months-NE) (12).

Prior to the FDA approval of capmatinib, there were no US-approved therapies that specifically targeted *MET*ex14 in NSCLC (4, 10, 14), but several other targeted therapies were given off-label to patients with *MET*ex14 NSCLC (15). These included crizotinib, a tyrosine kinase inhibitor with activity against *MET*, *ALK*, and *ROS1*; and cabozantinib, a tyrosine kinase inhibitor with activity against a broad range of targets, including *MET*, *VEGFR2*, *RET*, *FLT3*, and *KIT* (16). Reports have shown promising clinical activity for these agents in patients with *MET*ex14 NSCLC (2, 16, 17).

For lung cancer patients with *MET*ex14, broad molecular profiling, such as next-generation sequencing (NGS) testing, can be successfully used to guide treatment decisions (2, 16, 17). Studies have shown that broad molecular profiling, which allows patients to match with an appropriate therapy, can result in improved outcomes (18–20). NGS testing also saves personnel working time and consumables related to biomarker tests and reduces the overall cost of testing per patient (21, 22).

A potential barrier to use of targeted agents may be the time required for NGS testing to identify *MET*ex14 as well as other actionable biomarkers in patients with NSCLC (23), which may result in some patients initiating treatment with systemic therapies prior to receiving broad molecular testing results. Over the years, testing companies have tried to expedite the processing time for NGS testing, but the time needed to receive testing results can still be multiple weeks (23). Although NGS panels have longer testing turnaround times compared with single tests, NGS can reliably cover a broader spectrum of alterations (24). Patients with *MET*ex14 NSCLC have commonly been treated with chemotherapy or immuno-oncology (IO) therapy (9). Programmed death-ligand 1 (PD-L1) expression ≥50% has been detected in nearly half of patients with *MET*ex14 (25); however, as seen in patients with many other targetable oncogenic drivers, patients with *MET*ex14 tend to have generally poor responses to immunotherapy (25, 26), and the evidence of the clinical effectiveness of these treatments, particularly IO therapy, on patients with *MET*ex14 has been mixed, potentially due to differences in study populations, designs, and outcomes assessments (9, 27–29). Insights into real-world testing patterns and clinical outcomes associated with current treatments in the *MET*ex14 advanced NSCLC (aNSCLC) population may help physicians to make appropriate treatment choices for these patients. This study used a nationwide US de-identified clinico-genomic database (CGDB) to describe real-world biomarker testing patterns and assess the clinical outcomes of the most frequently used first-line (1L) and second-line (2L) therapies (IO monotherapy or chemotherapy) in patients with *MET*ex14 aNSCLC.

## Methods

### Study Design and Objectives

This was a descriptive retrospective study using electronic health records (EHR) and genomic data from the Flatiron Health (FH)–Foundation Medicine Inc (FMI) NSCLC CGDB to examine real-world biomarker testing patterns and clinical outcomes of chemotherapy and IO in patients with *MET*ex14 aNSCLC for treatment in 1L and 2L. The start of 1L therapy after aNSCLC diagnosis served as the index date and the beginning of the post-index period (follow-up), which extended until the earlier of 2 events: death or the last activity date (the last observed encounter during the patient’s follow-up).

### Data Source

Retrospective longitudinal clinical data were derived from EHR, comprising patient-level structured and unstructured data, curated *via* technology-enabled abstraction. During the study period, these data originated from approximately 280 cancer clinics (~800 sites of care) in the FH network, representing more than 2.4 million patients with cancer (30, 31). Genomic data were sourced from comprehensive genomic profiling (CGP) performed by FMI, with a genomic database of >400,000 sequenced tumor samples from patients in the US. The linking of patient data was performed by de-identified, deterministic matching using a third party in an institutional review board–approved, Health Insurance Portability and Accountability Act (HIPAA)-compliant fashion. Data collected from January 1, 2011, to December 31, 2019, were used in this study.

### Patient Selection

Participants first had to meet baseline criteria for the FH-FMI NSCLC CGDB, as follows:

- At least 2 documented clinical visits in the FH network on or after January 1, 2011, and on or before June 30, 2019
- International Classification of Diseases (ICD) code for lung cancer (ICD-9 162.x or ICD-10 C34x or C39.9); chart-confirmed diagnosis of NSCLC
- CGP test by FMI on or after the date of chart-confirmed diagnosis of NSCLC on a tumor sample (collected no earlier than 30 days before the FH diagnosis date) with pathologist-confirmed histology that is consistent with NSCLC
- Uniquely and deterministically matched demographic characteristics

Selected participants were eligible for inclusion if they were diagnosed with aNSCLC (initial diagnosis of stage IIIB, IIIC, IV, IVA, or IVB disease or diagnosis of early-stage NSCLC with subsequent development of recurrent or progressive disease) between January 1, 2011, and October 2, 2019 (patients diagnosed with aNSCLC with less than 90 days before data cutoff were not considered for analysis due to limited follow-up time); were treated with at least 1 line of therapy starting on or after January 1, 2011; were at least 18 years of age as of the index date; and had *MET*ex14 confirmed from a solid-tumor biopsy according to the FMI NGS report closest to the index date. Key exclusion criteria were a gap in structured activity after the advanced diagnosis date, defined as no records of vital information, medication administrations, non-canceled drug orders, or laboratory tests/results within 90 days after the advanced diagnosis date (as patients without observed activity during this timeframe may have been treated at a practice outside the FH network); evidence of treatment with a clinical trial drug in the 1L setting; and treatment with combination therapies of chemotherapy + IO or chemotherapy + MET inhibitor in the 1L setting.

### Outcome Measurements

The duration between the specimen collection date and aNSCLC diagnosis was assessed for both PD-L1 and FMI NGS testing (specimen collection date could have occurred before or after aNSCLC diagnosis). Because the physician order date was incomplete for PD-L1 testing and not available for FMI NGS testing, the duration from the specimen collection date until date of availability of the results for PD-L1 and FMI NGS testing was explored. Only patients with the same specimen collection date for PD-L1 and FMI NGS testing in 2019 (the most recent year for which data were available) were included in this analysis to minimize potential overestimation due to tissue archiving and cross-institutional bias. Note that molecular biomarker testing methods such as CGP and immunohistochemistry are often performed on specimens that had originally been obtained for a different purpose (e.g., diagnostic biopsy). Therefore, the duration from specimen collection to result availability reflects a combination of physician decision-making and testing time. We advise interpreting this duration with this caveat in mind.

Real-world progression-free survival (rw-PFS) and overall survival (OS) by 1L and 2L therapy class were assessed only among those who initiated chemotherapy or IO monotherapy as 1L treatment, because a limited number of patients initiated other therapies during the study period. Progression data were curated by abstractors from the EHRs of patients in the FH-FMI CGDB (32). Progression events were defined as distinct episodes in which the treating clinician concluded that there had been growth or worsening in the disease of interest. Such episodes are abstracted using an abstraction approach that uses clinician assessment as the main source of evidence, with radiology, laboratory, and pathology results as confirmatory documentation. Overall survival events were based on documented death dates. All other patients were censored on their last activity date in the database.

### Statistical Analysis

This study was descriptive in nature. 1L and 2L rw-PFS were estimated using a Kaplan-Meier analysis stratified by drug class (chemotherapy and IO monotherapy). An event for 1L or 2L rw-PFS was defined as the earlier of documented progression or death after the 1L or 2L treatment start date, respectively. Patients without an event were censored on their last clinic note date. Statistical analysis was performed using SAS version 9.4 (SAS Institute Inc).

### Ethics Approval and Consent to Participate

This study was designed, implemented, and reported in accordance with the Guidelines for Good Pharmacoepidemiology Practices (GPP) of the International Society for Pharmacoepidemiology (2016), the STROBE (Strengthening the Reporting of Observational Studies in Epidemiology) guidelines, and the ethical principles laid down in the Declaration of Helsinki.

FH-FMI processed data were de-identified according to the Expert Determination method as outlined in HIPAA to prevent re-identification of patient-level data to protect patients’ confidentiality. The CGDB data source and research activities were reviewed and approved by WCG IRB, the independent central institutional review board (IRB) of record for FH and FMI. Based on minimal-risk research, a waiver of informed consent and HIPAA authorization was approved by WCG IRB. WCG IRB holds full accreditation of their human subjects research program by the Association for the Accreditation of Human Research Protection Programs, Inc. In addition, WCG IRB of record assures the ethical conduct of research and compliance with federal, state, and institutional guidelines.

This study fulfills the criteria of a European Network of Centres for Pharmacoepidemiology and Pharmacovigilance (ENCePP) study and followed the ENCePP Code of Conduct (European Medicines Agency 2016).

## Results

### Patient Selection

**Figure 1:**
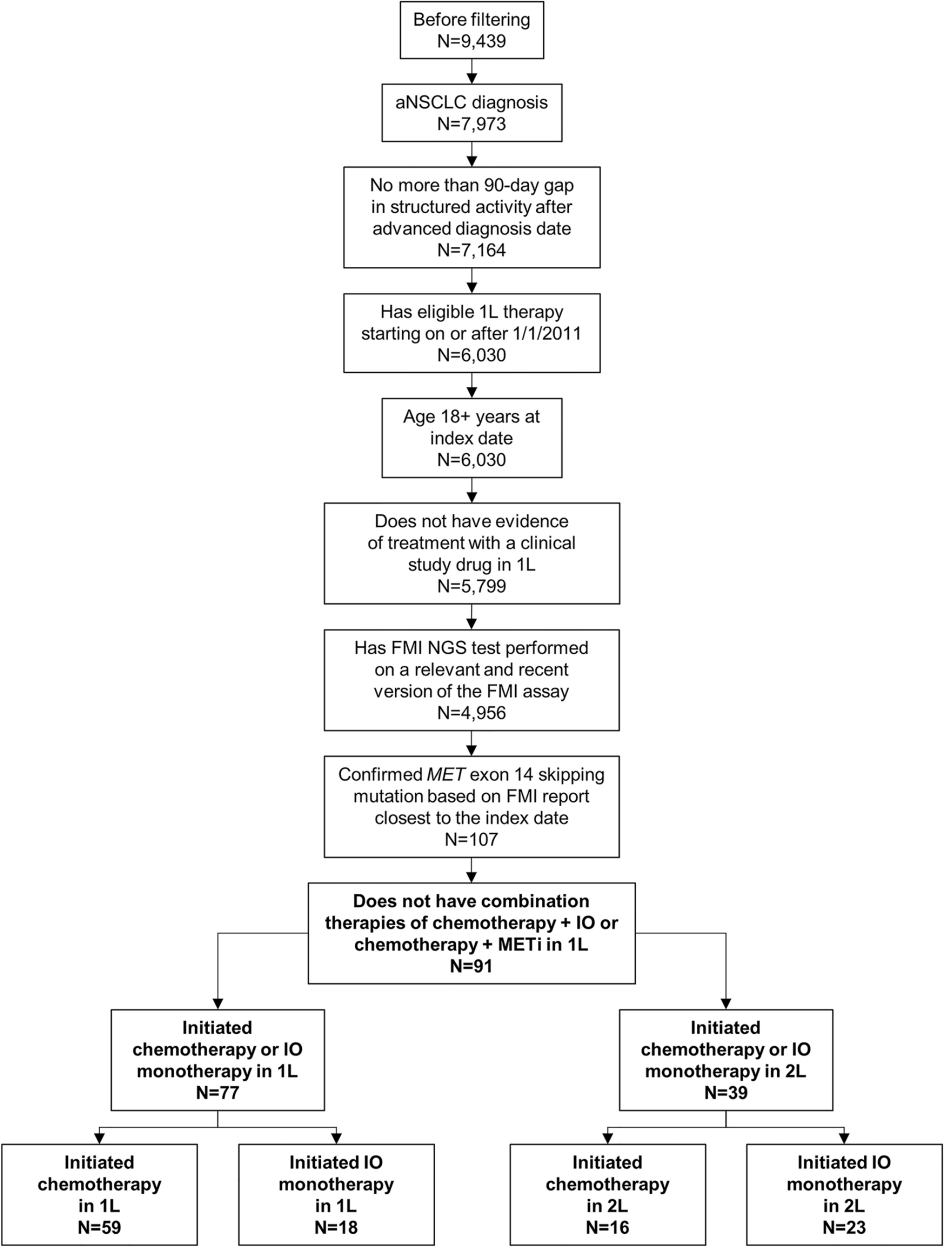
Patient Selection. Patients with non-small cell lung cancer (NSCLC) who were eligible for inclusion in the study were first required to meet baseline criteria for the Flatiron Health–Foundation Medicine Inc (FMI) NSCLC clinico-genomic database. Of those patients (N=9439), 91 also met the study-specific inclusion criteria and were included in the biomarker testing pattern analyses. Only patients who initiated chemotherapy or immuno-oncology (IO) monotherapy as first line (1L; n=77) or second line (2L; n=39) were included in clinical outcome analyses. aNSCLC, advanced non-small cell lung cancer; METi, MET inhibitor; NGS, next-generation sequencing.

Of the 9439 patients meeting baseline criteria for the NSCLC CGDB, 91 met all study-specific selection criteria and were included in the biomarker testing pattern analyses. Only patients who initiated chemotherapy or IO monotherapy as 1L or 2L were included in rw-PFS and OS analyses (**Figure 1**). Chemotherapy regimens consisted of carboplatin, cisplatin, docetaxel, etoposide, gemcitabine, paclitaxel, paclitaxel protein-bound, or pemetrexed, including combinations with afatinib, bevacizumab, and/or ramucirumab. IO therapies received were nivolumab, pembrolizumab, atezolizumab, and durvalumab. A total of 16 patients with confirmed *MET*ex14 were excluded due to initiation of combination therapies of chemotherapy and IO or chemotherapy and MET inhibitor in the 1L setting.

### Baseline Characteristics

Patient characteristics at baseline are shown in **Table 1**. The median age of patients at the index date was 75 years, with a range from 48 to 85 years. A high proportion of patients (80%, n=73/91) were initially diagnosed with stage III or IV NSCLC, representing a primarily *de novo* aNSCLC population. A total of 89% of patients were diagnosed with non-squamous cell carcinoma. Bone (42%) and brain (20%) were the most common sites of metastases at the index date. Other oncogenic drivers (i.e., *ALK* rearrangement, *BRAF* mutation, *EGFR* mutation, *KRAS* mutation, *ROS1* rearrangement) were rare in this patient population. Ninety-three percent of patients were managed in a community practice setting.

**Table 1:** Baseline characteristics.

Number (%) of patients	91 (100)
Age at index date, years^a^	
Median (IQR)	75.00 (70.00-80.00)
Mean (SD)	73.75 (7.32)
Range	48.00-85.00
Age at index date category, n (%)^a^	
35-49 years	1 (1)
50-64 years	12 (13)
65-74 years	31 (34)
75+ years	47 (52)
Sex, female, n (%)	52 (57)
Race, n (%)	
White	68 (75)
Asian	1 (1)
Black or African American	4 (4)
Other	10 (11)
Missing	8 (9)
Practice type, n (%)	
Academic	6 (7)
Community	85 (93)
Group stage at initial diagnosis, n (%)	
Stage I-II	15 (16)
Stage III	24 (26)
Stage IV	49 (54)
Missing	3 (3)
Time from initial diagnosis to advanced diagnosis, months	
Median (IQR)	0.03 (0.03-4.80)
Mean (SD)	4.29 (12.93)
Range	0.03-111.01
Histology, n (%)	
Non-squamous cell carcinoma	81 (89)
Squamous cell carcinoma	6 (7)
NSCLC histology NOS	4 (4)
ECOG performance status, n (%)^b^	
0	15 (16)
1	31 (34)
2	13 (14)
3	4 (4)
Missing	28 (31)
Concomitant mutations, n (%)	
* ALK* rearrangement positive	1 (1)
* BRAF* mutation positive	1 (1)
* EGFR* mutation positive	1 (1)
* KRAS* mutation positive	5 (5)
* ROS1* rearrangement positive	0 (0)
Year of index date, n (%)^a^	
2013	2 (2)
2014	11 (12)
2015	8 (9)
2016	23 (25)
2017	18 (20)
2018	15 (16)
2019	14 (15)
Follow-up time from index date to last activity, months^a^	
Median (IQR)	7.62 (4.47-22.21)
Mean (SD)	15.28 (16.38)
Range	0.03-67.98
First-line therapy class, n (%)	
Chemotherapy^c^	59 (65)
IO monotherapy^d^	18 (20)
MET inhibitor monotherapy^e^	11 (12)
Erlotinib	3 (3)
Sites of metastasis at index date, n (%)^a^ ^,^ ^f^	
Bone	38 (42)
Brain	18 (20)
Lung	14 (15)
Adrenal	14 (15)
Distant lymph node	12 (13)
Liver	9 (10)
Other	22 (24)

a Index date is the start of first-line therapy.

b Closest ECOG performance status 30 days prior to or 7 days after the index date.

c Chemotherapy: carboplatin, cisplatin, docetaxel, etoposide, gemcitabine, paclitaxel, paclitaxel protein-bound, or pemetrexed, including combinations with afatinib and/or bevacizumab.

d IO drugs: nivolumab, pembrolizumab, and atezolizumab; database search also included avelumab, cemiplimab, and durvalumab, but no patients in the study received these agents.

e MET inhibitor drugs: crizotinib and cabozantinib.

f Date of metastasis is provided at month-year granularity in the underlying data. Presence of metastases at any point prior to index date is defined as the presence of metastases prior to or in the same month and year as the index date.

ECOG, Eastern Cooperative Oncology Group; IO, immuno-oncology; IQR, interquartile range; NOS, not otherwise specified; NSCLC, non-small cell lung cancer.

### Testing Patterns

Out of 91 patients included in the study, 62% (n=56) had documentation of receiving PD-L1 testing. Among these patients, 39% (n=22/56) were tested through FMI, while 61% (n=34/56) were tested through external laboratories. These data may be underestimated, because patients could have received PD-L1 testing outside the FH network. PD-L1 status was ≥50% in 46% (n=26) of patients, 1% to 49% in 20% (n=11) of patients, negative or <1% in 29% (n=16) of patients, and missing/unknown in 5% (n=3) of patients.

**Figure 2:**
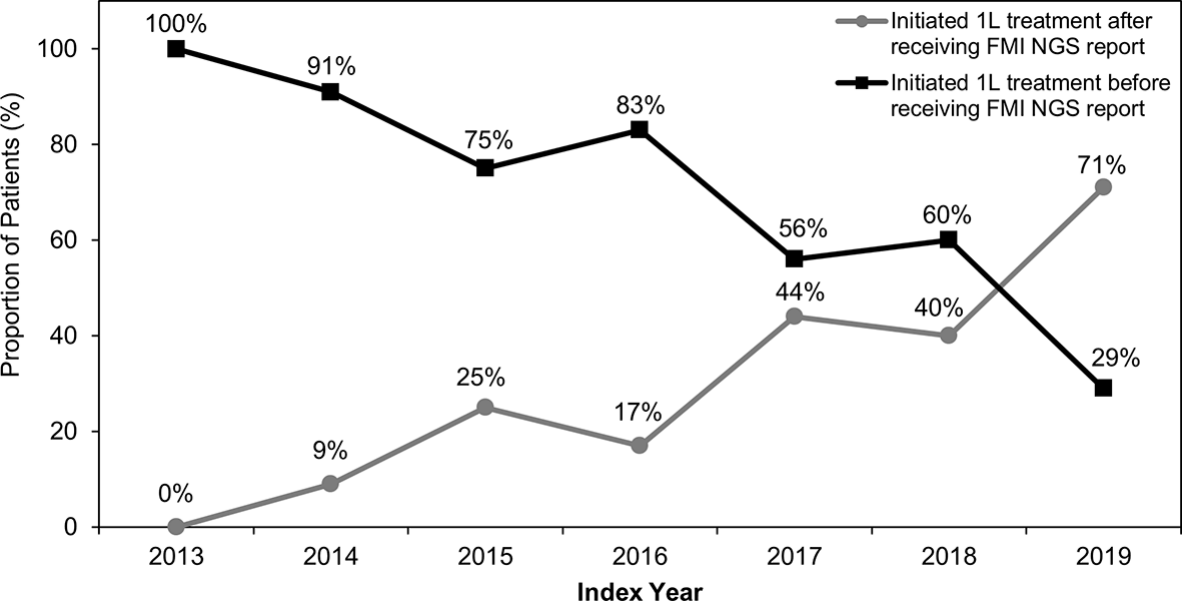
Treatment Initiation Relative to Receipt of FMI NGS Reports Between 2013 and 2019. Between 2013 and 2019, the percentage of patients who initiated first-line (1L) treatment before Foundation Medicine Inc (FMI) next-generation sequencing (NGS) testing reports were received generally decreased, while the percentage of patients initiating 1L treatment after receiving FMI NGS testing reports generally increased.

Overall, 34% (n=31/91) of patients received their FMI NGS testing reports before initiation of 1L therapy, whereas 66% (n=60/91) of patients received their reports after starting 1L therapy. Prior to 2019, most patients initiated 1L therapy before receiving their FMI reports (**Table 2** and **Figure 2**). Generally, between 2013 and 2019, there was a trend toward an increasing proportion of patients initiating 1L treatment after FMI NGS reports were received (**Figure 2**). Of patients with *de novo* stage IV NSCLC, 24% (n=12/49) received FMI NGS testing results before initiation of 1L treatment and 76% (n=37/49) received FMI NGS testing results after initiation of 1L treatment.

**Table 2:** Testing patterns relative to treatment initiation.

Year of index date	FMI NGS report before index date, n (%)	FMI NGS report after index date, n (%)
2013	0	2 (100)
2014	1 (9)	10 (91)
2015	2 (25)	6 (75)
2016	4 (17)	19 (83)
2017	8 (44)	10 (56)
2018	6 (40)	9 (60)
2019	10 (71)	4 (29)

FMI, Foundation Medicine Inc; NGS, next-generation sequencing.

### Specimen Collection and Testing Results

**Figure 3:**
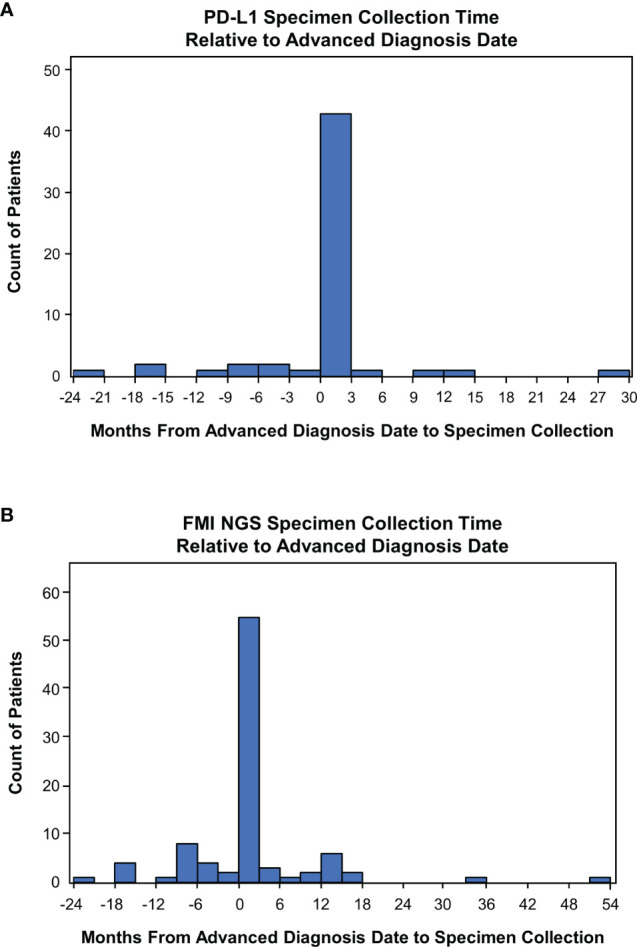
Time of Specimen Collection Relative to Advanced Non-Small Cell Lung Cancer Diagnosis. **(A)** Frequency histogram of PD-L1 testing times. **(B)** Frequency histogram of Foundation Medicine Inc (FMI) next-generation sequencing (NGS) testing times. Specimen collection times were defined as the duration from the date of advanced diagnosis to specimen collection.

For 84% (n=47/56) and 78% (n=71/91) of patients, a specimen was collected for PD-L1 and FMI NGS testing, respectively, on or after their advanced diagnosis date. Specimen collection occurred within 3 months post aNSCLC diagnosis for most patients (PD-L1: 43/56, 77%; FMI NGS: 55/91, 60%; **Figure 3**). For patients with the same PD-L1 and FMI NGS specimen collection date in 2019 (n=9), the median time from specimen collection to reported results for PD-L1 and FMI NGS testing was 20 days (interquartile range [IQR], 17-42 days) and 27 days (IQR, 27-54 days), respectively—a difference of 1 week (**Table 3**).

**Table 3:** Duration from specimen collection to reported results: same-day PD-L1 and FMI NGS specimen collection.

PD-L1^a^	
N	9
Duration, median (IQR), days	20 (17-42)
Duration, mean (SD), days	43 (56)
FMI NGS^b^	
N	9
Duration, median (IQR), days	27 (27-54)
Duration, mean (SD), days	63 (68)

a Duration from PD-L1 specimen collection to report availability, calculated as reported date − specimen collected date + 1.

b Duration from FMI NGS specimen collection to report availability, calculated as reported date − specimen collected date + 1.

FMI, Foundation Medicine Inc; IQR, interquartile range; NGS, next-generation sequencing.

For 73% (n=41/56) of patients tested for PD-L1, specimens were collected for both FMI NGS and PD-L1 tests on the same date (**Supplementary Table 1**). For 18% (n=10/56) of patients tested for PD-L1, samples were collected for PD-L1 testing prior to the collection of samples for FMI NGS testing.

### rw-PFS and OS

1L rw-PFS and OS were assessed in patients who initiated chemotherapy (n=59/77, 77%) or IO monotherapy (n=18/77, 23%) as 1L therapy and in patients who initiated chemotherapy (n=16/39, 41%) or IO monotherapy (n=23/39, 59%) as 2L therapy. 1L rw-PFS was not assessed for other therapy groups, because the sample sizes were inadequate for analyses (1L: MET inhibitor n=11, erlotinib n=3; 2L: MET inhibitor n=7, clinical study drug n=6, afatinib n=2, ceritinib n=1, crizotinib + pembrolizumab n=1). The most common 1L and 2L chemotherapy regimens were platinum doublets (platinum-based chemotherapy plus another chemotherapy agent), constituting 71% (n=42/59) and 50% (n=8/16) of patients, respectively. The most common 1L IO monotherapy regimen was pembrolizumab, constituting 83% (n=15/18) of patients. In the 2L setting, the most common IO monotherapy regimens were nivolumab and durvalumab, constituting 43% (n=10/23) and 30% (n=7/23) of patients, respectively.

**Figure 4:**
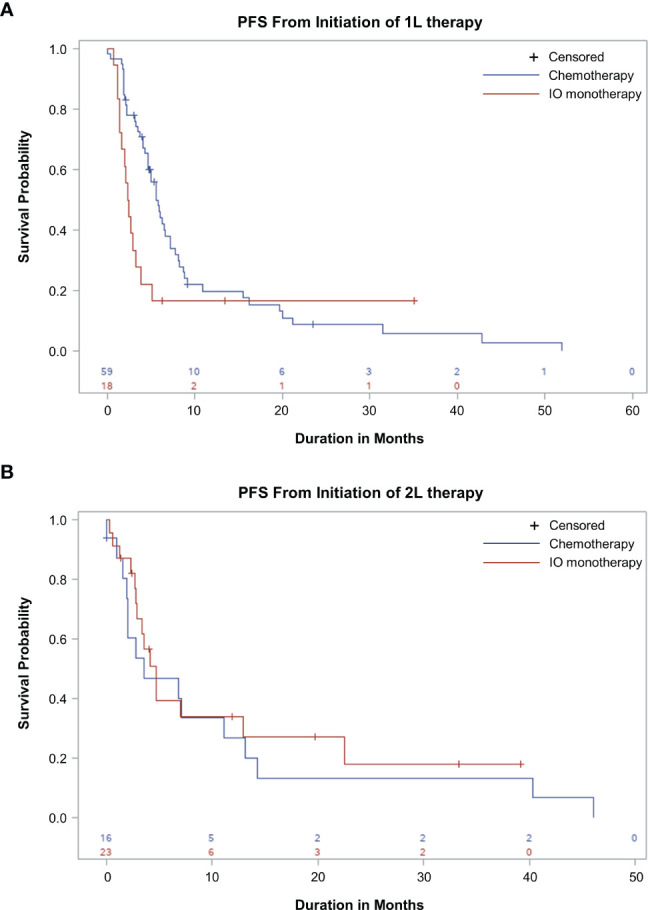
Real-World Progression-Free Survival (rw-PFS) With First-line (1L) and Second-line (2L) Therapy. Kaplan-Meier curves showing rw-PFS for patients treated with chemotherapy or immuno-oncology (IO) in 1L **(A)** or 2L setting **(B)**. The start of the analysis is the start of 1L therapy **(A)** and the start of 2L therapy **(B)**. This study is descriptive in nature, and comparisons between treatment groups should not be made.

In the 1L setting, median 1L rw-PFS was 5.7 months (95% CI, 4.6-7.1 months) and 2.4 months (95% CI, 1.4-3.2 months) in patients receiving chemotherapy and IO monotherapy, respectively (**Figure 4A**). The 3-, 6-, and 12-month rw-PFS rates for patients initiating chemotherapy in 1L were 78%, 46%, and 20%, respectively. For patients initiating IO in the 1L, the 3-, 6-, and 12-month 1L rw-PFS rates were 33%, 17%, and 17%, respectively. The median OS in the 1L setting was 20.0 months (95% CI, 10.9-24.6 months) and not reached (95% CI, 3.1 months-not reached) in patients receiving chemotherapy and IO monotherapy, respectively. The 3-, 6-, and 12-month OS rates for patients initiating chemotherapy in 1L were 95%, 82%, and 60%, respectively. For patients initiating IO in 1L, the 3-, 6-, and 12-month OS rates were 78%, 61%, and 52%, respectively.

In the 2L setting, median 2L rw-PFS was 3.5 months (95% CI, 1.9-11.1 months) and 4.7 months (95% CI, 2.8-12.9 months) in patients receiving chemotherapy and IO monotherapy, respectively (**Figure 4B**). The 3-, 6-, and 12-month 2L rw-PFS rates for patients initiating chemotherapy in 2L were 54%, 47%, and 27%, respectively. For patients initiating IO in 2L, the 3-, 6-, and 12-month 2L rw-PFS rates were 67%, 40%, and 34%, respectively. The median OS in the 2L setting was 15.3 months (95% CI, 4.0-41.1 months) and 19.3 (95% CI, 11.6 months-not reached) in patients receiving chemotherapy and IO monotherapy, respectively. The 3-, 6-, and 12-month OS rates for patients initiating chemotherapy in 2L were 87%, 73%, and 51%, respectively. For patients initiating IO in 2L, the 3-, 6-, and 12-month OS rates were 76%, 76%, and 70%, respectively.

For the limited number of patients initiating MET inhibitor monotherapy (n=11) or erlotinib (n=3) in 1L, the median 1L rw-PFS was 4.5 months (95% CI, 2.2 months-not reached) and 4.1 (95% CI, 0 months-not reached), respectively. Median OS for these patients was 10.0 months (95% CI, 4.9 months-not reached) and 24.7 months (95% CI, 0 months-not reached), respectively. However, no FDA-approved MET inhibitors were included in this study and these findings are based on a small number of patients; caution should be taken in interpreting these results.

## Discussion

The time associated with biomarker testing in aNSCLC is known as a critical operational issue impacting treatment decision-making (26); however, few studies have provided up-to-date assessments on this topic. We found only two studies in this domain (33, 34), neither of which looked at testing time for NGS in recent years. Our study utilized the most recent data available to provide contemporary assessment on the timing associated with biomarker testing (both PD-L1 and NGS) from collection of tumor specimen to receipt of test results among patients with *MET*ex14 aNSCLC. For patients with advanced cancer, the time needed for receiving NGS testing results may affect real-world treatment decisions regarding the adoption of targeted therapy. Importantly, NGS testing requires about 2 weeks for results and costs more than individual single-gene tests; however, the overall cost is lower and the time to all results is shorter compared with multiple sequential single-gene tests for all potentially actionable oncogenic drivers, which makes NGS testing a preferred method for comprehensive molecular profiling (22). It should be noted that the assessment of time from biopsy to receiving testing results, in relation to the subsequent clinical benefit of patients receiving various therapies, has not been well studied. This study examined real-world testing patterns for PD-L1 and FMI NGS testing and assessed the clinical outcomes with chemotherapy and IO monotherapy in patients with *MET*ex14 aNSCLC prior to capmatinib US approval.

Our results show that the time from specimen collection to reported results was shorter for PD-L1 testing than for FMI NGS testing in 2019, with a median difference of 1 week. Whether this arose from differences in clinical decision-making, characteristics of the 2 forms of testing, or some combination of these factors could not be ascertained with the data available. Additionally, the duration between specimen collection and report date in this study should not be interpreted as turnaround time, which is defined as the time between test order and report date; note that specimen collection could have occurred before or after the test order in real-world practice. In this study, based on a primarily community oncology setting in the US, we found that twice as many patients with *MET*ex14 initiated 1L therapy prior to receiving their FMI NGS reports as initiated 1L therapy after receiving their reports. Although multiple lung cancer guidelines recommend early comprehensive genomic profiling prior to systemic treatment initiation (4, 35, 36), our study showed that testing was often postponed until after the treatment decisions were made, and therefore the optimal clinical outcomes were not achieved. Our study sample was restricted to those who received NGS testing; if otherwise assessed in a broader sample of patients with NSCLC, it is expected that comprehensive biomarker testing may be further delayed or not completed. One of the primary reasons for this delay or lack of NGS testing, excluding cost, is the consideration around the additional waiting or turnaround time to obtain testing results. Because NGS testing turnaround time may be shortened due to advancement of testing technology in recent years, we attempted to further explore the most recent data, which showed a difference of only about 1 week between NGS testing and PD-L1 testing. In our analysis, we saw a trend toward more patients receiving an NGS report prior to treatment initiation in recent years, and fewer patients having the opposite order (**Table 2** and **Figure 2**). The shortened turnaround time and increased awareness of the importance of early NGS testing could be reasons leading to the positive shift with regard to testing pattern shown in **Table 2** and **Figure 2**. The data we presented here will further help physicians to rationalize the best treatment options for patients, as well as communicate to patients the importance of waiting for NGS testing results to achieve the best clinical outcomes. Furthermore, a few studies also support the idea of waiting in order to ensure that the most appropriate treatment is initiated first (19, 37, 38). Importantly, earlier treatment initiation for advanced NSCLC may not be associated with longer survival (37, 38), and a large retrospective study has shown that in patients with actionable oncogenic drivers, receiving a targeted therapy is associated with longer survival (19).

Our analysis of measures of effectiveness for chemotherapy or IO monotherapy in patients with *MET*ex14 demonstrated a median 1L rw-PFS of 5.7 months and 2.4 months in patients receiving 1L chemotherapy and 1L IO monotherapy, respectively, and a median 2L rw-PFS of 3.5 months and 4.7 months in patients receiving 2L chemotherapy and 2L IO monotherapy, respectively. Patients who received IO monotherapy in 1L showed 3- and 6-month 1L rw-PFS rates of 33% and 17%, respectively, indicating that most patients experienced rapid disease progression even with these treatments. Our analysis also found a median OS of 20.0 months in patients receiving 1L chemotherapy but not reached in patients receiving 1L IO monotherapy, respectively, and a median OS of 15.3 months and 19.3 months in patients receiving 2L chemotherapy and 2L IO monotherapy, respectively. These results are consistent with previous reports showing lower overall survival in patients with *MET*ex14 NSCLC receiving nontargeted therapies (chemotherapy and IO) relative to those receiving targeted therapies (9, 39). In a retrospective chart review study, patients who received treatment with a MET inhibitor had a median overall survival of 25.3 months, whereas patients who did not receive a MET inhibitor had a median overall survival of 10.9 months (39). In another retrospective review of stage IV NSCLC patients with *MET*ex14 comparing those who received a MET tyrosine kinase inhibitor in 1 or more lines of therapy (n=27) with those who did not (n=34), the median overall survival was 24.6 months and 8.1 months, respectively. Among patients who received crizotinib as their first MET inhibitor, the median PFS was 7.4 months (9). Patients in these studies could have received a MET inhibitor in any line of treatment, not just in 1L. A report from the Sarah Cannon Research Institute showed that patients with *MET*ex14 NSCLC were responsive to MET inhibitor therapy after receiving standard-of-care therapy in 1L (40).

A phase 2 study of capmatinib in patients with aNSCLC and confirmed *MET*ex14 (GEOMETRY mono-1) demonstrated substantial antitumor activity regardless of the line of therapy in which capmatinib was used. The median progression-free survival (PFS) was 12.4 months (95% CI, 8.2 months-NE) and 5.4 months (95% CI, 4.2-7.0 months) among treatment-naive patients in the 1L setting and previously treated patients in the 2L or third-line (3L) setting, respectively (11). A *post hoc* analysis of patients treated with capmatinib in the 2L or 3L setting in the GEOMETRY mono-1 trial reviewed responses in patients who did or did not receive IO in the 1L or 2L setting. Among patients who received prior IO, the median PFS was 8.3 months (95% CI, 4.2-12.6 months; data not mature). Among patients without prior IO, the median PFS was 5.4 months (95% CI, 4.2-6.9 months) (41). Furthermore, a recent analysis reviewed treatment-naive patients with *MET*ex14 aNSCLC treated with 1L capmatinib in the GEOMETRY mono-1 trial compared with a matched real-world cohort treated with 1L chemotherapy and/or immunotherapy. The median PFS was 12.0 months (95% CI, 5.5-20.7 months) and 6.2 months (95% CI, 3.4-9.1 months) for patients treated with 1L capmatinib or 1L chemotherapy and/or immunotherapy, respectively. Additionally, after left truncation was accounted for in the real-world dataset, the median overall survival was 20.8 months (95% CI, 12.6 months-not reached) and 14.8 months (95% CI, 9.0 months-not reached) for patients treated with 1L capmatinib or 1L chemotherapy and/or immunotherapy, respectively (31). Taken together, both real-world and clinical trial evidence highlights the need to test all patients with NSCLC for oncogenic driver mutations, including *MET*ex14, and the importance of early testing.

A retrospective review of 24 patients with *MET*ex14 NSCLC receiving single-agent (anti–PD-L1) or combination (anti–PD-1 and anti–CTLA-4) IO reported an ORR of 17%, with short DOR and PFS (28). In the 21 assessable patients, the median PFS was 1.9 months (95% CI, 1.7-2.7 months) (28). Furthermore, in the retrospective IMMUNOTARGET registry study, 23 patients with advanced *MET*ex14 treated with immunotherapy had a median PFS of 4.7 months (95% CI, 1.8-7.8 months) (26). These results are consistent with case reports showing limited efficacy of IO in patients with *MET*ex14 NSCLC (29, 42). In each of these 4 studies or reports, responses were limited even in tumors with high PD-L1 expression (26, 28, 29, 42). A recent study with a small sample size (N=13) by Mayenga et al, however, showed prolonged responses to 2L IO treatment in 6 patients with *MET*ex14 NSCLC previously treated with chemotherapy (N=13), with 5 patients showing a partial or complete response within the first 4 months (27). This study finding contradicts the results of all other studies with larger sample sizes, although it indicates that the response to IO in patients with *MET*ex14 is still not fully understood; results based on only 13 patients pose the risk of overinterpretation of the finding (27, 28). The importance of broad molecular testing for driver mutations is further underscored by evidence showing that patients harboring driver mutations may not respond well to IO, in the case of genes such as *EGFR* and *ALK* (26, 43–45), or may have only modest responses, in the case of *BRAF* (26, 46). Accordingly, the National Comprehensive Cancer Network Clinical Practice Guidelines in Oncology for NSCLC now considers the presence of oncogenes that predict lack of benefit from single-agent IO or combination IO-chemotherapy a contraindication to the use of these agents (4). In particular, the guidelines note that patients with *MET*ex14 and high PD-L1 expression do not generally respond to IO (4).

Our study found that pembrolizumab and other PD-L1 inhibitors and chemotherapies were used frequently as 1L treatments in this patient population during the study time frame (January 1, 2011, to December 31, 2019). Although newly approved MET inhibitors will likely change the treatment landscape, the clinical outcomes associated with IO or chemotherapy in this study are still very much relevant and reflective of the current outcomes associated with these regimens. In our study, a small number of patients received IO plus chemotherapy or MET inhibitors as 1L treatments during the study period; however, due to the limited number of patients receiving these treatments, we are unable to provide data on the clinical outcomes with these treatment regimens.

### Limitations

This was an observational study using retrospective data. As such, differences in endpoints with small sample sizes should be interpreted with caution, and this study was not designed to provide comparisons between treatments. Data for this study were obtained from the EHRs of a network of oncology practices; any treatments or outcomes that occur outside of these practices may not be captured in the EHR system and thus are unaccounted for in this study.

Analyses for this study were based upon data available within the FH-FMI NSCLC CGDB. Given this constraint, testing times in our analysis reflected time from specimen collection to the reported test results, which does not reflect true testing turnaround time from the physician order date of the test to the reporting of results. This difference would necessarily be increased in patients whose samples may be sequentially tested and patients whose specimen was collected prior to aNSCLC diagnosis. In addition, cross-institutional bias may be introduced for testing not performed by FMI, such as PD-L1 immunohistochemistry testing routinely performed by institutions.

Furthermore, these analyses did not examine the effect of PD-L1 levels or tumor mutational burden (TMB) on rw-PFS. These biomarkers have been shown to be predictive of responses to IO monotherapy in non–oncogene-driven NSCLC (47, 48); however, patients with *MET*ex14 tend to have low TMB. Furthermore, in a retrospective analysis of patients with *MET*ex14 NSCLC, responses to IO monotherapy were modest compared with an unselected patient population, and responses were not enriched in the subset of patients with either high PD-L1 expression or high TMB (28).

Ultimately, the quality of information extracted from real-world data depends on the quality of information available in the data source. Although the dataset has the potential for missing, inaccurate, or incomplete data, having access to source genomic data and technology-enabled abstraction by specially trained human abstractors using documented policies and procedures and defined quality assurance and control activities aims to reduce data issues and increase completeness.

### Conclusions

This study attempted to explore real-world biomarker testing patterns of PD-L1 and FMI NGS among patients with aNSCLC with *MET*ex14. We observed a difference in median duration between specimen collection and reporting of PD-L1 and FMI NGS results of 1 week from a small sample of the original cohort. Nontargeted therapies (chemotherapy and IO) were the most frequently used 1L and 2L therapies, although they demonstrated limited clinical benefit in patients with *MET*ex14 aNSCLC. The findings of our study may prompt physicians to order NGS testing earlier to initiate appropriate treatment and achieve optimal clinical outcomes when treating aNSCLC with *MET*ex14. Future studies are needed to assess the testing turnaround time of NGS testing in the real-world setting and evaluate the effectiveness of targeted therapies, such as MET inhibitors, in the real-world clinical setting.

## Data Availability Statement

The data for this study are available from Flatiron, but restrictions apply to the availability of these data, which were used under license for the current study and so are not publicly available. Data are, however, available from the authors upon reasonable request and with permission of Flatiron.

## Ethics Statement

The studies involving human participants were reviewed and approved by WCG IRB. Written informed consent for participation was not required for this study in accordance with the national legislation and the institutional requirements.

## Author Contributions

FAZVK was involved in the conceptualization, investigation, formal analysis, and supervision. BC was involved in the conceptualization, investigation, formal analysis, and supervision. HK was involved in the investigation and formal analysis. JK was involved in the investigation and formal analysis. VD was involved in the conceptualization and formal analysis. SZ was involved in the formal analysis. MS was involved in the conceptualization and formal analysis. All authors were involved in the writing, review, and editing of the manuscript. All authors read and approved the final manuscript.

## Conflict of Interest

FAZVK is an employee of Novartis Pharma AG. BC, VD, SZ, and MS are employees of Novartis Pharmaceuticals Corporation. HK and JK are employees of Genesis Research.

The authors declare that this study received funding from Pharmaceuticals Corporation, East Hanover, NJ, USA. The funder had the following involvement with the study: Employees of the company were involved in medical accuracy review.

## Publisher’s Note

All claims expressed in this article are solely those of the authors and do not necessarily represent those of their affiliated organizations, or those of the publisher, the editors and the reviewers. Any product that may be evaluated in this article, or claim that may be made by its manufacturer, is not guaranteed or endorsed by the publisher.

## Abbreviations

1L, first-line; 2L, second-line; aNSCLC, advanced non-small cell lung cancer; CGDB, clinico-genomic database; CGP, comprehensive genomic profiling; DOR, duration of response; ECOG, Eastern Cooperative Oncology Group; EHR, electronic health record; FDA, US Food and Drug Administration; FMI, Foundation Medicine Inc; HIPAA, Health Insurance Portability and Accountability Act; ICD, International Classification of Diseases; IO, immuno-oncology; IQR, interquartile range; *MET*ex14, *MET* exon 14 skipping mutation; NE, not estimable; NGS, next-generation sequencing; NOS, not otherwise specified; NSCLC, non-small cell lung cancer; OS, overall survival; rw-PFS, real-world progression-free survival.
