# Supplementary material for: Biomarker Testing Patterns and Treatment Outcomes in Patients With Advanced Non-Small Cell Lung Cancer and MET Exon 14 Skipping Mutations: A Descriptive Analysis From the US
Source: Front Oncol. 2022 Feb 25;12:786124. doi: 10.3389/fonc.2022.786124 (PMC8915293; doi:10.3389/fonc.2022.786124)
Supplement: Supplementary file 1 [file Table_1.docx]

***Supplementary Material***

**Supplementary Table 1. PD-L1 Testing Specimen Collection Date Relative to FMI NGS Specimen Collection Date.**

|  | **N (%)** |
| --- | --- |
| Not tested for PD-L1 | 35 (38) |
| Tested for PD-L1 | 56 (62) |
| PD-L1 specimen collection date < FMI NGS specimen collection date | 10 (18) |
| PD-L1 specimen collection date = FMI NGS specimen collection date | 41 (73) |
| PD-L1 specimen collection date > FMI NGS specimen collection date | 5 (9) |

FMI, Foundation Medicine Inc; NGS, next-generation sequencing.
